# Comparing the Effects of Duo-Functional Triple-Layer Films Enriched with Different Sources of Curcumin on the Shelf-Life of Fish

**DOI:** 10.3390/foods13213499

**Published:** 2024-10-31

**Authors:** Joanna Tkaczewska, Piotr Kulawik, Nikola Nowak, Wiktoria Grzebieniarz, Paweł Krzyściak, Wondyfraw Tadele, Eskindir Endalew Tadesse, Rafał Szram, Paulina Guzik, Ewelina Jamróz

**Affiliations:** 1 Department of Animal Product Technology, Faculty of Food Technology, University of Agriculture, ul. Balicka 122, 30-149 Kraków, Poland; piotr.kulawik@urk.edu.pl (P.K.); wontade09@gmail.com (W.T.); eskindire.tadesse@gmail.com (E.E.T.); rafal.szram@urk.edu.pl (R.S.); paulina.guzik@urk.edu.pl (P.G.); 2 Department of Chemistry, Faculty of Food Technology, University of Agriculture, ul. Balicka 122, 30-149 Kraków, Poland; nikola.nowak@urk.edu.pl (N.N.); wiktoria.grzebieniarz@urk.edu.pl (W.G.); ewelina.jamroz@urk.edu.pl (E.J.); 3 Department of Infection Control and Mycology, Faculty of Medicine, Jagiellonian University Medical College, ul. Czysta 18, 31-121 Kraków, Poland; pawel.krzysciak@uj.edu.pl; 4 Department of Packaging and Logistics Processes, Cracow University of Economics, Rakowicka 27, 31-510 Kraków, Poland

**Keywords:** interactions between active compounds, film matrices, furcellaran

## Abstract

The objective of this study was to compare the effects of two types of active triple-layer films containing curcumin on the shelf-life of salmon fillets. One film (Film A) contained pure curcumin dissolved in lemongrass essential oil, while the other (Film B) incorporated curcumin from *Curcuma longa* extract dissolved in citral. The impact of these active films on the preservation of salmon fillets quality and safety was studied by analyzing factors such as color parameters, sensory evaluation, lipid oxidation, and biogenic amines. Despite good active properties measured in vitro, both films harmed the sensory quality and color of salmon. However, the incorporation of active ingredients in biopolymer films has shown the potential to inhibit biogenic amine formation. The findings pave the way for future research to explore the synergistic effects of combining various natural extracts with active packaging films.

## 1. Introduction

The demand for innovative food packaging solutions has increased significantly in recent years, driven by the need for enhanced food safety and extended shelf-life. Among these innovations, multilayer biopolymer films have emerged as a promising alternative due to their environmentally friendly properties [1]. These films are composed of environmentally non-toxic biopolymers, making them fully biodegradable. What sets them apart is their ability to incorporate active or smart ingredients, enabling the development of packaging materials that not only extend the shelf-life of products but also indicate the degree of spoilage [2].

However, despite encouraging outcomes observed in laboratory settings, there are still several limitations hindering the widespread adoption of biopolymer films. One of the most significant challenges is their relatively high gas permeability and lower mechanical properties compared to their synthetic counterparts. Many endeavors to modify biopolymer films have been documented in the literature, but a common stumbling block is the limited availability of active ingredients [1]. To overcome this limitation, a promising approach is the use of multilayered biopolymer films. These films incorporate multiple layers of biopolymers or their complexes, enabling the unrestricted inclusion of active ingredients without concerns about antagonistic effects. This approach allows for full utilization of the biopolymers’ functional capabilities [1,2]. By addressing these challenges, multilayer biopolymer films hold great potential for enhancing food packaging and preservation, while remaining environmentally friendly.

Curcumin has emerged as a subject of keen interest in recent years, primarily owing to its remarkable antimicrobial and antioxidant properties [3]. These properties make it a promising candidate for enhancing the shelf-life of perishable products like fish. Moreover, curcumin offers an additional advantage as a marker due to its propensity to undergo a color change as it oxidizes over time. This dual functionality of curcumin, both as a preservative and a visual indicator of freshness, underscores its potential significance in food packaging materials.

Despite the recognized benefits of curcumin, a notable gap exists in the literature concerning its integration into multilayer film systems. Multilayer films represent a sophisticated approach to packaging, offering enhanced barrier properties and versatility compared to traditional monolayer films. By incorporating curcumin into these multilayer films, it is possible to explore novel avenues for extending the shelf-life of perishable foods like fish. Additionally, such research can contribute to addressing the shortcomings inherent in biopolymer films, thereby paving the way for more effective and sustainable packaging solutions. Thus, the exploration of curcumin within multilayer film systems holds considerable promise as an innovative strategy to overcome current limitations in food packaging technology.

There is a pressing need to test active biopolymer films on real food matrices as part of the development and validation process. While in vitro studies provide valuable initial insights, they are conducted under controlled laboratory conditions that cannot fully replicate the complex and dynamic environment of real food systems. Real food matrices present unique challenges, such as varying pH levels, moisture content, and interaction with other food components, which can significantly impact the performance of active films. Testing in real food environments allows for a comprehensive assessment of how these factors influence the functionality of the films, ensuring they perform effectively under practical conditions. Furthermore, the antimicrobial and antioxidant properties of active films need to be validated in real food systems to confirm their efficacy in extending shelf life and maintaining food quality [3].

In our previous research, two types of innovative triple-layer films were designed and studied. In the initial investigation [4], pioneering triple-layer films were effectively crafted by combining gelatin hydrolysate, furcellaran, and chitosan. Within the composite, an ethanolic extract of curcumin, along with lemongrass essential oil, was integrated into the middle layer (Film A). The outcomes were encouraging, signifying the material’s substantial antimicrobial and intelligent capabilities. Nevertheless, it is worth noting that the films exhibited a notable drawback, as they displayed elevated permeability to gases.

In the second investigation, efforts were made to enhance the performance characteristics of triple-layer films composed of furcellaran, gelatin, and the active ingredient *Curcuma longa* extract in the additional citral component (Film B) [5]. This research marked a significant breakthrough as it involved the preparation of a curcumin extract by dissolving curcumin in citral, resulting in a compound with robust antimicrobial and antioxidant properties. Citral played a dual role in this context, serving both as an effective antimicrobial agent and as a potent solubilizer for curcumin in conjunction with ethanol. Subsequently, this curcumin extract, derived from curcuma powder with the assistance of citral and ethanol, was incorporated into the middle layer of the biopolymer film. The matrix for this layer was furcellaran, renowned for its well-documented gel-forming properties and suitability as a matrix for active ingredients. To address the issue of high gas permeability, a furcellaran–gelatin complex was implemented as the third layer to reduce the film’s permeability parameters to gases. The resulting film was characterized in terms of its functional properties, which proved to be very promising. Given the potent antimicrobial and smart properties of both curcumin and citral, it is recommended to further investigate their active and intelligent capabilities through in vitro and in vivo analyses.

Therefore, this study aims to compare the active effects of two different types of triple-layer films enriched with different sources of the active compound curcumin, one enriched with pure curcumin in lemongrass oil (Film A) and the other with *Curcuma longa* extract in citral (Film B), during the storage of a model fish product—Atlantic salmon. This work aims to indicate how the appropriate selection of biopolymers and the preparation of the active ingredient can affect the final usefulness of the packaging material.

## 2. Materials and Methods

### 2.1. Materials

In the work, for the produced films, the main ingredients were furcellaran (Est-Agar AS, Karla village, Estonia). According to the manufacturer’s specifications, furcellaran had a molecular weight of 2.95 × 10^5^ g/mol and contained 79.61 g of carbohydrates, 1.18 g of protein, and 0.24 g of fat in 100 g of the product. Low molecular weight chitosan (90% deacetylation degree) was procured from Pol-Aura (Zabrze, Poland).

TWEEN 80 was bought at Chempur (Piekary Slaskie, Poland). Salmon fillets were purchased in a mega store (Makro Cash and Carry, Krakow, Poland). Gelatin hydrolysates from carp skins were obtained via the method described in a previous study by Tkaczewska et al. [6]. All of the other chemical reagents used in this study were obtained from Sigma-Aldrich (Sigma-Aldrich GmbH, Munich, Germany) and were of analytical grade.

Film A contained pure curcumin (Pol-Aura, Zabrze, Poland -Cat. No.: A147405; purity 98 %; M_w_ 368.38) dissolved in lemongrass oil (VitaFarm Company, Lipno, Poland), while Film B contained an extract from *Curcuma longa* (Kwizda, Linz, Austria), serving as a source of curcumin, incorporated in citral (All Organic Treasures GmbH, Wiggensbach, Germany).

### 2.2. Film Preparation

For this research, two kinds of triple-layer films were used. The first film (Film A) was created as described by Jamróz, Cabaj, Tkaczewska, Kawecka, Krzyściak, Szuwarzyński, Mazur, and Juszczak [4]. It constituted triple-layered, pH-sensitive, and active films based on furcellaran (FUR), chitosan (CHIT), and gelatin hydrolysates (HGEL). The 120 mg of curcumin was dissolved in 30 mL of ethyl alcohol. This mixture was then placed in an ultrasonic bath at 35 °C for 20 min. Following this, 1 mL of lemongrass essential oil and 1 mL of TWEEN 80 were added to the clear solution to stabilize the emulsion.

The second film (Film B) was made according to the method described by Nowak, Grzebieniarz, Juszczak, Cholewa-Wójcik, Synkiewicz-Musialska, Huber, Touraud, Kunz, and Jamróz [5]. To obtain the curcumin extracts, the powdered rhizomes were extracted using a ternary solvent mixture of citral, ethanol, and water (68:27:5) in centrifuge tubes at a 1:4 powder-to-solvent ratio. The extractions were conducted at room temperature, stirring the mixtures at 1300 rpm for 1 h. Subsequently, the mixtures were centrifuged at 4200× *g* for 10 min and the solution was decanted into a fresh vessel to separate the plant remnants from the extract. These triple-layer films based on furcellaran and gelatin are made with a middle layer containing different concentrations of *Curcuma longa* extract with the addition of citral. *Curcuma longa* extract in citral was 3 different levels of concentration (1 mL/250 (CUR 1), 2 mL/250 mL (CUR 2), and 3 mL/250 mL (CUR3) of film forming solution. *Curcuma longa* extract was prepared by dissolving curcuma powder in a citral-ethanol solution, following the methodology developed in our previous study. The citral acted as a solubilizer and an antimicrobial agent, enhancing the properties of the *Curcuma longa* extract. The use of the FUR/GEL layer was intended to reduce water vapor permeability, while citral was used as the main active ingredient in lemongrass oil. 

### 2.3. Active Properties of the Obtained Films

Due to the antimicrobial and antioxidant properties of FILM A being researched and published [4], only an analysis of the active properties of FILM B was performed.

#### 2.3.1. Antimicrobial Properties

The antimicrobial properties were investigated against Gram-positive cocci (*Staphylococcus aureus* ATCC 25922, Enterococcus faecalis ATCC 29212), glucose-fermenting Gram-negative bacilli (Escherichia coli ATCC 25923, Salmonella enterica BAA664), non-fermenting Gram-negative bacilli (*Pseudomonas aeruginosa* ATCC 27869), and yeasts (*Candida krusei* ATCC 6258, *Candida albicans* ATCC 10231). This study was conducted following previously published methods Jamróz et al. [7]. Briefly, film samples measuring 1-cm × 1-cm were aseptically placed onto 10 mL agar medium. Muller Hinton 2 agar was used for bacteria, while Sabouraud Dextrose Agar was used for yeasts. A standardized microorganism suspension was prepared by diluting 100 mL of a 0.5 McFarland inoculum per 10 mL. This suspension was poured onto the film samples, and the medium was allowed to solidify. Subsequently, incubation was carried out at 37 °C for 24 h.

#### 2.3.2. Antioxidant Properties

The antioxidant properties of the produced films were evaluated using two complementary methods: the ferric reducing antioxidant power (FRAP) assay and the DPPH radical scavenging activity test. For the FRAP assay, the ability of the films to reduce Fe^3+^ ions was described in the method by Behbahani et al. [8]. Aqueous extracts of the films were produced by placing 150 mg of film in 10 mL of distilled water at 50 °C. Then, the mixture was placed in a shaking water bath at 50 °C for 10 min. During this time, a FRAP solution was prepared using 300 mM of acetic buffer (pH 3.6), 20 mM FeCl_3_–3H_2_O, and 20 mM solution of 2,4,6-tripyridyl-s-triazine in hydrochloric acid (10 mM TPTZ in 40 mM HCl). Afterward, the FRAP reagent was mixed with the aqueous film extracts in a volume ratio of 0.4:3.6, and all elements were incubated in the dark for 10 min at 37 °C. After this time, the absorbance value was measured at 593 nm using the Helios Gamma UV-1601 spectrophotometer (Thermo Fisher Scientific, Waltham, MA, USA). In addition to the FRAP assay, the DPPH radical scavenging activity of the films was measured. An aqueous film extract was mixed with a solution of DPPH in ethanol, at a ratio of 0.2:2.8 (0.1 mM). Subsequently, the blend was allowed to incubate for 10 min at ambient temperature, shielded from light. After the incubation period, the absorbance was gauged at 517 nm by employing the Helios Gamma UV-1601 spectrophotometer (Thermo Fisher Scientific, Waltham, MA, USA). Distilled water was used in the blank instead of the film extract. 

### 2.4. Assessing the Impact of the Film on the Quality of the Fish Product

#### 2.4.1. Preparation of Samples

Salmon (*Salmo salar*) fillets, each weighing 100 g, were fully wrapped with either Film A or Film B, ensuring that the entire surface of the fillets was covered. To ensure consistency in fat content and guarantee the comparability of experimental data, the same central part of each fillet was selected for all samples. Additionally, some of the samples were wrapped only in synthetic cling wrap (LDPE) to serve as a positive control. For further comparison, unwrapped fish samples were used as a second control group (C). All samples were placed in transparent PET food containers with matching transparent lids and stored under refrigerated conditions (~4 °C) for 14 days. For each analysis, three samples were randomly selected from each group on days 0, 4, 8, 11, and 14 of storage.

#### 2.4.2. Color of the Salmon Fillets

The analysis used the CR 200 Minolta Chroma Meter (Konica Minolta, Osaka, Japan), employing the CIE Lab* scale. Each fillet sample underwent three readings on two opposite sides of the equatorial region for comprehensive analysis.

#### 2.4.3. Effect of Tested Films on the Safety of Fish During Storage

##### Microbiological Analysis of Salmon Fillets

The tests were conducted in accordance with ISO 6887:1:2017 standards. The evaluations encompassed the determination of the total microorganism count utilizing the flood method on Plate Count agar (Biomaxima, Warsaw, Poland), with an incubation period of 48 h at 30 °C. Additionally, the yeast and mold counts (YM) were analyzed via surface inoculation on DRBC agar (Biomaxima, Poland), with an incubation duration of 120 h at 25 °C.

##### Biogenic Amine Analysis of Salmon Fillets

The analysis encompassed various biogenic compounds, specifically, nine amines (histamine, cadaverine, putrescine, spermine, spermidine, 2-phenylethylamine, agmatine, tyramine, and trimethylamine) along with ammonia levels. Each sampling day involved testing three distinct fish specimens, with duplicate measurements performed for each sample (3*n* × 2). The biogenic amine profile was evaluated through a high-performance liquid chromatography (HPLC) protocol adapted from the methodology described in the literature. The samples underwent a derivatization process employing benzoyl chloride as the primary derivatizing agent, following established protocols. The chromatographic analysis was conducted using a Shimadzu LC-10VP system (Shimadzu, Kyoto, Japan), which incorporated several key components: a UV/VIS detection unit (Spectra-Physics SP 8450, Analytical Inc., Manchester, UK) and a specialized low-pressure gradient pump system (Shimadzu LC-10ATVP) integrated with a quaternary mixer (Shimadzu FVC-10ALVP). The separation process was accomplished using a C18 reverse-phase column (nucleosil, 250 × 4.6 mm, 5 mm particle size) manufactured by Mecherey-Nagel (Duren, Germany).

##### Assessing the Extent of Lipid Oxidation Through the TBARS Method

A portion of 10 g was collected from the thoroughly homogenized material. The extraction procedure involved combining the sample with 30 mL of perchloric acid (4%) and adding 0.75 mL of ethanolic BHT solution (4%). Following homogenization, the mixture underwent centrifugation (RCF = 10,733× *g*) for a duration of 10 min. The resulting supernatant was processed through Whatman No. 1 filter paper and collected in a 50-mL volumetric vessel, with subsequent volume adjustment using 4% perchloric acid. The analysis continued by combining equal volumes (4 mL each) of the prepared extract and thiobarbituric acid solution (0.02 M TBA). This mixture was subjected to thermal treatment in a water bath maintained at 90 °C for 60 min, followed by a cooling period. The spectrophotometric measurement was performed using a Helios Gamma instrument (Thermo Fisher Scientific, Essex, UK), with absorbance readings taken at 532 nm. A 4% perchloric acid solution served as the reference blank

##### Sensory Analysis

Ten trained panelists, all regular consumers of salmon, conducted sensory analyses. They assessed the odor, color, and texture of the salmon fillets using a 5-point descriptive scale, ranging from 1 (representing the poorest quality description) to 5 (representing the finest description of quality attributes), with 3 serving as the acceptability threshold. Additionally, panelists assigned an overall product quality score using a hedonic scale ranging from 1 to 9 points. It is worth noting that only raw samples were evaluated, so taste was not considered in the assessment.

### 2.5. Statistical Analysis

Statistical analysis was conducted utilizing Statistica v13.0 software (Tibco, Palo Alto, CA, USA). The normality of the results and homogeneity of variances were analyzed using Saphiro–Wilk and Levene’s test, respectively. In the case of active properties of the films, a one-way analysis of variances was performed with the Tukey post-hoc test for distinguishing the differences between groups. In the case of analyses performed on model food products, a two-way analysis of variances was performed with independent variables being the film type and time of storage. The confidence interval used was α = 0.05. 

## 3. Results and Discussion

### 3.1. Antioxidant Properties

The antioxidant properties of the obtained Film B were analyzed using two different antioxidant mechanisms, including the ability to reduce iron ions in the third oxidation state via the FRAP method and the radical scavenging activity. Various types of Film B were examined, each containing different concentrations of the active compound from *Curcuma longa* extract in citral: CUR 1 at a concentration of 1 mL/250 mL of film-forming solution, CUR 2 at 2 mL/250 mL, and CUR 3 at 3 mL/250 mL. A control film without the addition of *Curcuma longa* extract was also evaluated. The antioxidant activity of Film A has been presented in our previous publication [4].

**Figure 1:**
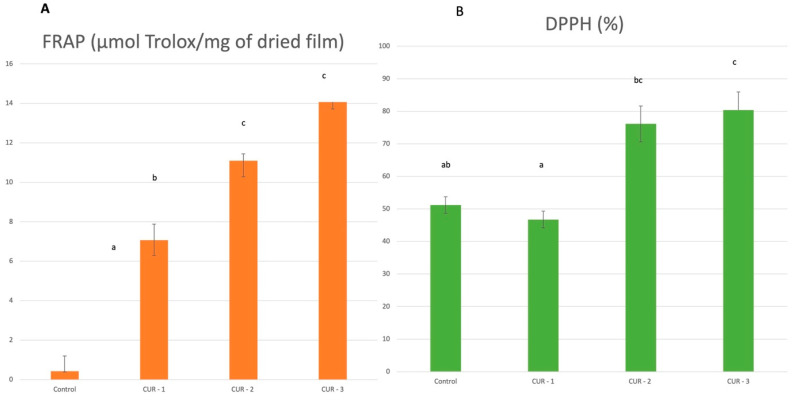
Antioxidant activity of active films with curcumin extract in citral. Control—a film without *Curcuma longa* extract in citral; CUR 1—*Curcuma longa* extract in citral at a concentration of 1 mL/250 mL of film-forming solution; CUR 2—*Curcuma longa* extract in citral at a concentration of 2 mL/250 mL of film-forming solution; CUR 3—*Curcuma longa* extract in citral at a concentration of 3 mL/250 mL of film forming solution. Values are expressed as mean ± SD. Letters indicate significant differences (*p* < 0.05).

For the control film, the lowest antioxidant activity using the FRAP and DPPH methods was observed (Figure 1). 

The antioxidant potential of the composites was significantly increased by adding the active ingredient. Compared to the control film, a 32-fold increase in FRAP values and an almost 30-percent higher DPPH value for the CUR-3 sample were observed. In comparison to the results obtained in the previous study [4], with the films containing curcumin in lemongrass oil (Film A), the FRAP value was improved from 0.52 ± 0.09 to 14.07 ± 0.35 μmol Trolox/mg (film containing the highest concentration of the active ingredient), and a result of 80.39% ± 5.53 was achieved for the DPPH value, versus 49.80% ± 0.62 obtained in the previous study. These results indicate that the modification of the biopolymer matrix and the incorporation of curcumin in citral had a beneficial effect on the antioxidant properties of the multilayer films.

Curcumin is probably responsible for the increased antioxidant properties. The antioxidant action of curcumin is due to the breakdown of the phenolic chain and the donation of the H atom regarding its phenolic group. The antioxidant activity of curcumin in DPPH assays is due to a sequential proton loss and electron transfer mechanism [9]. No antagonistic effect of curcumin and citral was noted, and the combination of the two components significantly improved the antioxidant properties measured by FRAP and DPPH compared to the control film. The antioxidant activity of citral is due to its ability to break radical chains free by donating a hydrogen atom and due to scavenging free radicals [10]. 

It has been demonstrated by the study that the addition of curcuma extract to biopolymer films can significantly enhance their antioxidant properties, making them potentially useful in applications where protection against oxidation is crucial, such as in food packaging. The higher the concentration of curcumin, the better the antioxidant performance, making it an effective additive for such purposes.

### 3.2. Antimicrobial Activity

In previous work, a triple-layer film with curcumin extract enriched with lemongrass oil (FILM A) did not show an antimicrobial effect on the tested bacteria or fungi [4]. This behavior was attributed to the fact that the extract was placed in the middle layer and the 24-h analysis was insufficient to observe antimicrobial activity. This study investigated the antimicrobial activity of FILM B with three different CUR concentrations. While the tested films did not exhibit antimicrobial properties against Escherichia coli, Enterococcus faecalis, or Salmonella enterica, they showed antimicrobial activity (Table 1) against certain bacteria, such as *Pseudomonas aeruginosa* and *Staphylococcus aureus*, as well as fungi, including *Candida krusei* and *Candida albicans*. Incorporating the *Curcuma longa* extract with the addition of citral in the middle layer already resulted in an antimicrobial effect during the 24-h analysis, indicating that the first layer of film (furcellaran) was not an obstacle to the released active ingredient. Based on earlier studies [5], it can concluded that in the case of FILM A, the first layer of film (furcellaran + gelatin hydrolysate) was a better barrier to the curcumin extract enriched with lemongrass oil, as evidenced by the presented results. 

In previous works, both the antimicrobial effects of *C. longa* [11] and citral [12] extracts have been demonstrated. The antibacterial effect of curcumin involves inhibiting the function of filamentous temperature-sensitive mutant Z, which is a protein necessary for bacterial cell division [13]. However, the antimicrobial effect of citral results from the presence of α, β-unsaturated aldehydes, which behave like alkylating agents. Citral leads to changes and penetration of the lipid and protein structure in the bacterial cell wall and then leads to cell lysis and death [14]. The results indicate that FILM B, enriched with *Curcuma longa* extract and citral, demonstrates some antimicrobial activity, suggesting its potential applicability for packaging perishable food products to enhance food safety and shelf life.

### 3.3. Evaluating the Film’s Influence on Fish Product Quality

The influence of both types of films, FILM A and FILM B, on the quality of fish products is compared and evaluated. The performance of these films will be analyzed to determine their effectiveness in preserving the freshness, sensory attributes, and overall quality of the fish during storage. This comparative analysis aims to provide insights into how each film contributes to the preservation of fish products, highlighting their respective advantages and limitations.

#### 3.3.1. Color Parameters of Fish Fillets Covered with Films

Color is among the most critical factors influencing consumers’ food choices [15]. In Table 2, the color attributes of salmon fillets covered by different types of films are listed in different films during a 14-day storage period.

Surface yellowness is caused by the use of film. This effect was much more pronounced in fillets wrapped in film A than in film B, as evidenced by the presented results (Section 3.3.3). The fish fillets wrapped in film B demonstrated a lower b* value than fish wrapped in film A on days 8 and 11 of storage (Table 1). The increase in parameter b in the samples covered with film A can be related to the fact that curcumin was not bound in the film but was released onto the surface of the product. Bojorges et al. [16] observed similar results in their research. These authors also found an increase in parameter b for chicken samples covered with film with a curcumin addition. In their works, Zhang et al. [17] explain that adding herb extracts to films increases the yellowing of fresh meat packed in them.

#### 3.3.2. Effect of Tested Films on the Safety of Fish During Storage

##### Microbial Quality of Fish Products

**Figure 2:**
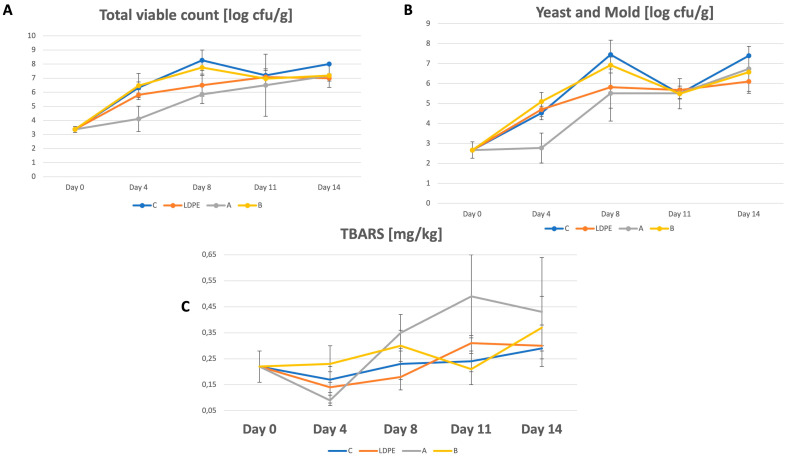
Microbial presence in fillets during the storage at 4 °C. (**C**)—samples without any films; LDPE-samples covered by LDPE films; (**A**)—sample covered with A films; (**B**)—sample covered with B films.

The Total Viable Count (TVC), as well as yeast and mold count of salmon fillets wrapped with and without synthetic and active films during storage for 14 days, is shown in Figure 2. 

The Total Viable Count (TVC) of all samples at the beginning of the experiment was 3.36 log CFU/g, suggesting the initial presence of microorganisms, potentially introduced during fillet preparation, packaging, or other stages. The TVC of the control sample (unwrapped) notably rose over the 14-day storage period, reaching approximately 8.00 log CFU/g by day 14. Only treatment with Film A resulted in the inhibition of microbiological growth, especially in the first stages of storage till day 8. From day 11, the antimicrobial effect could not be observed for any of the analyzed groups, and the final microbial concentrations of samples wrapped in CUR films were similar to the contamination of samples wrapped in LDPE films. 

Microbial growth has been reported to be retarded by films incorporated with chitosan and essential oils [18]. However, no microbial growth inhibition was observed in fillets covered with B films, which contain only curcumin and citral as an active ingredient without the addition of chitosan. While curcumin does have antimicrobial properties in vitro, its efficacy in food packaging films can be limited by factors such as concentration, release rate, environmental conditions, and interactions with food components [19]. 

Over the entire evaluation period, yeast and mold counts exhibited an upward trend in all groups assessed. Similar to the Total Viable Count (TVC), only film A demonstrated inhibition of yeast and mold growth on the fourth day of storage compared to the other treatments. Starting on the 6th day of storage, there were no differences noted about the amount of yeast and mold in any of the tested groups. Özvural et al. [20] argued that the encapsulation and binding of plant extracts or other active components in the coating could impede its antifungal effectiveness. Additionally, the release rates and behavior of antimicrobial substances are contingent upon several factors, encompassing polymer varieties, the methodology and procedure of film fabrication, film microarchitecture, interactions between antimicrobial agents and polymers, and environmental and medium-specific conditions. Moreover, there are many factors influencing the microbiological spoilage rate of fish [21]. That is why further research should be carried out to explain the interactions of the tested films with the model fish product.

##### Biogenic Amine Concentration in Salmo Salar Fillets Covered by Films

In fish products, biogenic amines related to deterioration are primarily produced by amino acid decarboxylases via contaminating microorganisms. Therefore, changes in quantities of biogenic amines are closely linked to the safety and quality of seafood. In the present experiment, all study groups resulted in an increasing trend of biogenic amine accumulation across the storage period (Table 3).

No statistically significant differences were observed between the tested groups on the fourth day of storage. On the eighth day of storage, samples covered with films A and B had significantly lower contents of tryptamine, 2-phenylalanine, putrescine, and histamine compared to the control groups (covered with food wrap and without any film). On the 14th day of storing the sample, only the histamine content remained significantly lower in the groups covered with film A (105.26 mg/kg). No statistical differences in histamine content were noted in the remaining groups. Histamine is an indicator of fish quality. Higher levels of histamine cause nausea, vomiting, diarrhea, an oral burning sensation or peppery taste, hives, itching, red rash, and hypotension [22]. The legal critical limits of histamine content in fish products vary depending on the country from 50 up to 400 mg/kg [23].

Tyramine can cause high levels of intoxication from seafood and fishery products. Tyramine was initially detected in the control samples on day 0 at 6.45 mg/kg and reached a final level between 32.82 and 34.08 mg/kg on day 14 (sample without active films). In contrast, the treated samples contained 26.47 and 26.76 mg tyramine/kg at the end of storage. The permittable limit of tyramine in marine products is 100 mg/kg [24]. Özogul et al. [25] stated that rosemary and sage tea extract prevent fish spoilage and decrease tyramine formation in sardines during storage. In the current study, the antimicrobial properties of curcumin could have possibly played a major role in preventing tyramine formation in samples during storage [26].

By examining the effect of gelatin-chitosan films with Maillard peptides, the analyzed contents of biogenic amines were comparable to the results obtained by Jiang et al. [27], reporting a level of histamine between 59.47 and 94.54 mg/kg on day 10 of storage at 4 (±1) °C in blue tuna slices. Elsabagh et al. [28] studied the combination effect of chitosan with different plant extracts on biogenic amine reduction in tuna fillets. The author showed that chitosan films combined with beetroot, curcumin, and garlic extracts caused histamine to remain at a level lower than 50 mg/kg in the samples. Furthermore, among the treated samples, chitosan incorporated with curcumin showed the lowest increase in biogenic amine formation. 

The obtained results in the presented study also confirm that curcumin extract, both in films with (Film A) and without chitosan (Film B), had an inhibitory effect on the formation of biogenic amines. However, this effect was most noticeable during the initial period of storage of fish fillets (day 8). The effect of inhibiting the formation of biogenic amines was not maintained during further storage when film B was used.

##### Lipid Oxidation

During storage, a significant increase in lipid oxidation was observed in all study groups (Figure 2C).

The results of the present study indicate that film B did not inhibit lipid oxidation in fish compared to the control samples. However, it has been found that this film also does not promote the oxidation process as is the case with film A. As demonstrated in our previous research [1], multilayer curcumin films promote lipid oxidation in a model fish product. An explanation for this phenomenon may be the fact that curcumin contains high levels of different polyphenols. Transition metals found in certain polyphenols can contribute to their prooxidant behavior. This occurs when these metals reduce metal ions, initiating redox-cycling and facilitating the formation of hydroxyl radicals via Fenton and Fenton-like reactions [29]. Consistent findings were obtained in a prior investigation [30] where an active film with high antioxidant potency was utilized as a preservative for food model products. These films inadvertently promoted oxidation in tofu, likely due to the presence of polyphenols sourced from protein hydrolysates and extracts from soybean bran, which were incorporated into the films. 

In salmon meat, no statistically significant influence of the packing in film B was observed on TBARS. The TBARS value for the fish fat during the early storage period was 0.22 mg/kg, and after 14 days, it reached values of 0.29, 0.30, and 0.37 mg/kg for the control, synthetic film, and study groups (film B), respectively. Incorporating *Curcuma longa* extract into active films enhanced antioxidant activity without impacting its ability to prevent lipid oxidation in fish products through refrigerated storage. These outcomes might be explained by the delayed release of active molecules from the film matrix or by the interactions formed between the film matrix and the active substance, thereby limiting their efficacy in vivo [31]. De Abreu et al. [32] suggested that augmenting the antioxidant concentration in the film leads to a more pronounced protective effect against oxidation. It could be hypothesized that elevating the curcumin content in the film might enhance its efficacy in preventing lipid oxidation in food products. However, additional research is warranted to validate this assumption.

Completely different results were obtained in the research oof Bojorges, Ríos-Corripio, Hernández-Cázares, Hidalgo-Contreras, and Contreras-Oliva [16]. They developed an edible alginate-based film infused with turmeric as an active ingredient and assessed its antioxidant capacity for use in fresh chicken breast and pork beef loin. Their findings suggest that the turmeric extract effectively combated lipid oxidation in all tested meats during refrigerated storage by reducing TBARS formation in the samples. The author attributes the inhibitory effect on meat oxidation to turmeric’s ability to obstruct oxygen and its antioxidant properties, particularly its high curcuminoid content. This observation is supported by other researchers, who noted that incorporating curcumin into nanoemulsion coatings decreased TBARS values in chickens, potentially inhibiting MDA formation.

Correspondingly, Fernandes et al. [33] suggest that incorporating natural extracts abundant in phenolic compounds helps to postpone lipid oxidation in meats.

The results obtained in our study indicate that the mere inclusion of ingredients rich in polyphenols among biopolymer films does not guarantee a delay in the oxidation of the meat packed in them. The method of applying active ingredients to the film and the form in which they are added determines the active in vitro effect.

#### 3.3.3. Sensory Evaluation

Sensory characteristics, including smell, meat color, texture, and overall score of the fish fillet, were compared in the control and treated packaging. The sensory evaluation demonstrated a significant decrease in acceptance in all samples over the course of storage duration (Table 4).

Based on the results, it can be noticed that all types of films, including LDPE, resulted in reduced odor scores. All groups were scored below the acceptance limit (3.0) already after 4 days of storage. Compared to the control treatment, the color and texture of salmon fillets on days 4 and 8 and the overall score on day 4 were significantly affected by the presence of films with the curcumin extract (films A and B). The color of the fillets was unacceptable after just 4 days of storage, while the color of LDPE-packed and control samples was still acceptable on the 8th day. In the case of overall scores, only LDPE-packed fish samples were deemed acceptable on day 4. No statistical differences were observed between groups in the final stage of sample storage (days 11 and 14).

The sensory assessment outcomes typically align with microbial and chemical analyses [22]. Due to the presence of high lipid oxidation and microbial growth, both the control samples and experimental groups of salmon fillets exhibited spoilage characteristics, including off-odor, sliminess, and discoloration after 4 days of storage. The antioxidant and antimicrobial properties of the films did not demonstrate effectiveness in mitigating spoilage effects, thereby failing to extend the shelf-life of the product while preserving its quality. The addition a curcumin extract to the film did not enhance the beneficial effects on color and overall acceptability of the fish in the final days of storage. Moreover, negative sensory evaluations of fillets wrapped in the tested films result mainly from the yellowing of the fish surface. This color comes from curcumin, which migrates from the film to the product under the influence of water contained in the fish. The bright yellow color mainly comes from fat-soluble polyphenolic pigments known as curcuminoids [34]. 

Other authors [35] concluded that fish treated with biodegradable films and natural preservatives presented better or similar sensory characteristics than the control treatment, which is not supported by our results. 

## 4. Conclusions

In conclusion, the use of duo-functional triple-layer films enriched with different sources of curcumin does not appear to offer a significant improvement in prolonging the shelf life of fish products, as their effects on lipid oxidation and microbial growth were limited, despite their potential to inhibit biogenic amine formation. Additionally, the negative impact of these films on the sensory properties of the stored fish highlights another challenge. To enhance the effectiveness of these films in food packaging applications, further research is necessary to optimize their permeability and the release rates of active compounds. The form and method of incorporating active ingredients into the films significantly influence their in vivo activity, suggesting that more attention should be given to these factors in future studies. Moreover, investigating the potential synergistic effects of combining different natural extracts with active packaging materials could lead to more advanced solutions that not only maintain food quality but also extend shelf life. Finally, further long-term research is warranted to validate the performance of these films in various food matrices and real-world storage conditions.

## Figures and Tables

**Table 1:** Antimicrobial activity of FILM B with curcumin extract at different concentrations.

	CUR-1	CUR-2	CUR-3	C
*Pseudomonas aeruginosa*	no/poor effect	partial effect	positive impact	no impact detected
*Staphylococcus aureus*	no impact detected	no impact detected	slightl effect (reduced growth near the film)	no impact detected
*Candida krusei*	no impact detected	positive impact	very good effect with broad inhibition zone	no impact detected
*Candida albicans*	partial/good effect	positive impact	positive impact with broad inhibition zone	no impact detected

CUR 1—*Curcuma longa* extract in citral at a concentration of 1 mL/250 mL of film-forming solution; CUR 2—*Curcuma longa* extract in citral at a concentration of 2 mL/250 mL of film-forming solution; CUR 3—*Curcuma longa* extract in citral at a concentration of 3 mL/250 mL of film forming solution, C—control film without *Curcuma longa* extract.

**Table 2:** Color parameter of salmon fillets covered by films.

		L*	a*	b*
C	Day 0	46.25 ^abcd^ ± 3.45	12.28 ^a^ ± 3.08	11.45 ^ab^ ± 3.85
C	Day 4	50.62 ^de^ ± 2.55	10.51 ^a^ ± 1.51	12.10 ^ab^ ± 1.77
LDPE		50.08 ^cde^ ± 3.62	11.19 ^a^ ± 1.59	12.51 ^abc^ ± 1.19
A		49.50 ^cde^ ± 6.02	11.93 ^a^ ± 1.74	16.51 ^bc^ ± 6.45
B		45.45 ^abc^ ± 3.23	9.27 ^a^ ± 2.80	15.82 ^abc^ ± 6.09
C	Day 8	47.57 ^abcde^ ± 2.63	12.35 ^a^ ± 2.54	12.82 ^abc^ ± 1.53
LDPE		51.31 ^def^ ± 3.79	10.87 ^a^ ± 1.93	11.22 ^ab^ ± 1.79
A		56.08 ^f^ ± 3.20	11.39 ^a^ ± 3.45	22.44 ^d^ ± 8.83
B		45.05 ^abc^ ± 3.23	10.36 ^a^ ± 1.33	12.94 ^abc^ ± 3.75
C	Day 11	51.90 ^ef^ ± 3.74	10.94 ^a^ ± 2.75	12.32 ^ab^ ± 2.32
LDPE		51.77 ^def^ ± 2.65	11.85 ^a^ ± 3.00	13.07 ^abc^ ± 2.05
A		49.70 ^cde^ ± 5.60	12.06 ^a^ ± 4.45	18.04 ^cd^ ± 6.99
B		43.85 ^ab^ ± 4.72	9.72 ^a^ ± 3.34	10.62 ^a^ ± 3.22
C	Day 14	48.56 ^bcde^ ± 2.50	11.86 ^a^ ± 1.37	10.54 ^a^ ± 1.61
LDPE		51.99 ^ef^ ± 3.43	10.62 ^a^ ± 2.81	11.72 ^ac^ ± 2.59
A		49.40 ^cde^ ± 6.68	11.62 ^a^ ± 3.26	13.43 ^abc^ ± 6.16
B		42.72 ^a^ ± 3.66	9.14 ^a^ ± 2.09	11.96 ^ab^ ± 5.65

Values are expressed as mean ± SD. Letters indicate significant differences (*p* < 0.05), C—sample without film, LDPE—sample cover with LDPE foil, A—sample cover with film A, B—sample cover with film B. Despite evident sensory differences in color, caused by the yellowing of the surface of salmon fillets through direct contact with curcumin, such a clear trend is hard to observe based on the instrumental color analysis. No significant difference (*p* > 0.05) was observed in the value of the L* parameter determining the lightness of color on the 4th and 8th storage days of the tested salmon fillets. However, a significantly lower color brightness was found in the subsequent days of storage regarding the samples covered with film B, compared to the remaining groups under study (L* 43.85 and 42.72 on the 11th and 14th days of storage).

**Table 3:** Biogenic amine contents in salmon filets during storage (mg/kg).

		Tryptamine	Phenylethylamine	Putrescine	Cadaverine	Histamine	Tyramine	Spermidine	Spermine
C	Day 0	0.03 ^a^ ± 0.00	0.39 ^a^ ± 0.19	3.21 ^a^ ± 0.86	21.78 ^a^ ± 4.37	33.15 ^a^ ± 11.61	6.45 ^a^ ± 2.32	2.76 ^ab^ ± 0.39	1.55 ^a^ ± 0.17
C	Day 4	0.00 ^a^ ± 0.00	1.01 ^a^ ± 0.12	5.48 ^a^ ± 0.44	36.81 ^bc^ ± 6.35	97.00 ^b^ ± 16.51	19.47 ^bcd^ ± 2.72	2.49 ^ab^ ±0.54	1.79 ^a^ ± 0.52
LDPE		0.00 ^a^ ± 0.00	1.25 ^b^ ± 0.31	5.89 ^a^ ± 0.94	34.40 ^b^ ± 4.75	117.32 ^bc^ ± 12.04	22.60 ^cd^ ± 1.00	2.23 ^ab^ ±0.64	1.37 ^a^ ± 0.43
A		0.01 ^a^ ± 0.00	0.54 ^a^ ± 0.22	3.41 ^a^ ± 1.82	23.62 ^a^ ± 9.17	50.02 ^a^ ± 19.93	12.97 ^ab^ ± 4.84	2.18 ^ab^ ±0.58	1.47 ^a^ ± 0.48
B		0.00 ^a^ ± 0.00	0.57 ^a^ ± 0.15	3.12 ^a^ ± 0.31	30.06 ^ab^ ± 6.34	49.76 ^a^ ± 11.94	13.12 ^ab^ ± 2.57	2.25 ^ab^ ±0.18	1.66 ^a^ ± 0.37
C	Day 8	2.10 ^d^ ± 0.69	5.11 ^d^ ± 0.65	18.51 ^de^ ± 1.44	48.70 ^d^ ± 2.48	218.47 ^g^ ± 54.60	32.25 ^e^ ± 4.52	2.56 ^ab^ ± 1.16	1.80 ^a^ ± 0.94
LDPE		1.27 ^c^ ± 0.44	8.44 ^e^ ± 1.76	13.72 ^cd^ ± 0.68	50.01 ^d^ ± 3.54	186.36 ^efg^ ± 19.08	33.78 ^de^ ± 1.14	2.13 ^ab^ ± 0.31	1.58 ^a^ ± 0.65
A		0.96 ^bc^ ± 0.11	3.06 ^bc^ ± 1.08	8.32 ^ab^ ± 0.42	45.98 ^cd^ ± 0.73	147.21 ^cde^ ± 14.61	27.05 ^e^ ± 4.12	1.78 ^a^ ± 0.25	1.02 ^a^ ± 0.20
B		0.46 ^ab^ ± 0.09	1.81 ^ab^ ± 0.67	6.03 ^a^ ± 2.03	29.29 ^ab^ ± 10.20	126.87 ^bcd^ ± 9.70	17.07 ^bc^ ± 6.70	1.76 ^a^ ± 0.53	1.03 ^a^ ± 0.29
C		2.10 ^d^ ± 0.65	5.81 ^d^ ± 1.25	19.92 ^e^ ± 4.04	46.61 ^cd^ ± 1.02	190.24 ^fg^ ± 22.70	32.82 ^e^ ± 6.31	2.10 ^ab^ ± 0.18	1.78 ^a^ ± 0.36
LDPE	Day 14	1.49 ^cd^ ± 0.47	9.43 ^e^ ± 1.93	18.69 ^de^ ± 2.15	48.74 ^d^ ± 0.65	164.40 ^def^ ± 18.79	34.08 ^e^ ± 2.75	1.84 ^a^ ± 0.31	1.55 ^a^ ± 0.52
A		1.45 ^cd^ ± 0.41	4.71 ^cd^ ± 1.19	31.68 ^f^ ± 7.73	46.88 ^cd^ ± 6.10	105.26 ^b^ ± 14.37	26.47 ^de^ ± 7.73	4.00 ^b^ ± 3.03	1.20 ^a^ ± 0.43
B		1.03 ^bc^ ± 0.28	5.25 ^d^ ± 0.87	11.38 ^bc^ ± 1.44	45.66 ^cd^ ± 4.65	158.36 ^cdef^ ± 8.60	26.76 ^de^ ± 4.09	2.24 ^ab^ ± 0.45	1.35 ^a^ ± 0.31

Letters indicate significant differences (*p* < 0.05), C—samples without any films; LDPE—samples covered by LDPE films; A—sample covered with A films; B—sample covered with B films.

**Table 4:** Sensory analysis of salmon fillets covered by films.

		Smell	Meat Color	Texture	Overall Score
C	Day 0	5.00 ^f^ ± 0.00	5.00 ^e^ ± 0.00	5.00 ^f^ ± 0.00	9.00 ^f^ ± 0.00
C	Day 4	2.42 ^de^ ± 0.77	4.41 ^e^ ± 0.93	3.70 ^ef^ ± 0.65	3.06 ^e^ ± 1.80
LDPE		2.39 ^de^ ± 0.92	4.22 ^e^ ± 0.62	4.22 ^f^ ± 0.46	5.17 ^e^ ± 1.64
A		2.81 ^e^ ± 0.94	2.03 ^abcd^ ± 1.09	2.69 ^cd^ ± 1.14	3.19 ^cd^ ± 1.60
B		2.06 ^cd^ ± 0.86	1.89 ^abcd^ ± 0.76	2.44 ^cd^ ± 1.00	2.67 ^bc^ ± 1.37
C	Day 8	1.77 ^ab^ ± 1.19	3.30 ^d^ ± 1.01	3.27 ^d^ ± 0.86	2.79 ^bc^ ± 1.01
LDPE		2.50 ^cde^ ± 0.87	3.33 ^d^ ± 0.90	3.53 ^de^ ± 1.14	4.13 ^de^ ± 1.68
A		2.87 ^de^ ± 0.90	1.70 ^ab^ ± 0.77	2.27 ^abc^ ± 1.21	2.00 ^cabc^ ± 0.43
B		2.37 ^cde^ ± 0.48	2.57 ^bcd^ ± 0.94	3.03 ^cd^ ± 1.16	3.04 ^bc^ ± 0.78
C	Day 11	1.30 ^ab^ ± 0.59	2.50 ^cd^ ± 1.49	1.97 ^bc^ ± 0.85	2.23 ^abc^ ± 1.79
LDPE		1.07 ^a^ ± 0.26	2.27 ^bcd^ ± 1.43	1.57 ^ab^ ± 0.73	1.90 ^abc^ ± 1.28
A		1.60 ^abc^ ± 0.47	1.37 ^ab^ ± 0.55	1.33 ^ab^ ± 0.36	1.63 ^abc^ ± 0.58
B		1.90 ^bcd^ ± 0.71	1.40 ^ab^ ± 0.34	1.90 ^abc^ ± 0.57	2.20 ^abc^ ± 0.82
C	Day 14	1.00 ^a^ ± 0.00	1.80 ^abc^ ± 0.68	1.40 ^ab^ ± 0.51	1.00 ^a^ ± 0.00
LDPE		1.00 ^a^ ± 0.00	1.47 ^ab^ ± 0.64	1.33 ^ab^ ± 0.49	1.10 ^a^ ± 0.28
A		1.07 ^a^ ± 0.26	1.33 ^ab^ ± 0.41	1.13 ^ab^ ± 0.30	1.17 ^ab^ ± 0.31
B		1.03 ^a^ ± 0.13	1.13 ^a^ ± 0.23	1.10 ^a^ ± 0.21	1.00 ^a^ ± 0.00

Values are expressed as mean ± SD. Letters indicate significant differences (*p* < 0.05), C—sample without film; LDPE—sample cover with LDPE foil; A—sample cover with film A; B—sample cover with film B.

## Data Availability

The raw data supporting the conclusions of this article will be made available by the authors on request.
